# The Volunteer Satisfaction Survey (VSS): Adaptation and Psychometric Properties among Portuguese Volunteers

**DOI:** 10.3390/ejihpe13010002

**Published:** 2022-12-30

**Authors:** Cátia Martins, Saúl Neves de Jesus, José Tomás da Silva, Conceição Ribeiro, Cristina Nunes, Francisca Ferreira Cunha, Beatriz Marcelo

**Affiliations:** 1 Psychology Research Centre (CIP), Universidade do Algarve, Campus de Gambelas, 8005-139 Faro, Portugal; 2 Research Centre for Tourism, Sustainability and Well-Being (CinTurs), Universidade do Algarve, Campus de Gambelas, 8005-139 Faro, Portugal; 3 Centre for Social Studies, Faculty of Psychology and Educational Sciences, University of Coimbra, 3000-115 Coimbra, Portugal; 4 Centre of Statistics and its Applications (CEAUL), Instituto Superior de Engenharia, Universidade do Algarve, Campus da Penha, 8005-139 Faro, Portugal

**Keywords:** volunteer satisfaction, volunteering, functionalist approach, psychometric analysis

## Abstract

(1) Background: Volunteering satisfaction is one significant construct that nourishes the sustaining of volunteer work, and it is present in reference models such as the three-stage volunteer process model (VPM). The volunteer satisfaction survey (VSS), created by Vecina, Chacón and Sueiro, evaluates three different domains of volunteer satisfaction: specific motivations, organization management and volunteering tasks. The aim of this study was to adapt the instrument and explore the psychometric properties of the 17 items of the VSS in a sample of Portuguese volunteers. (2) Methods: The sample was composed of 335 Portuguese volunteers (aged between 14 and 81 years), mainly women (76.4%). Measures included volunteer satisfaction, work engagement and organizational commitment. (3) Results: The original three-factor model was tested with a confirmatory factor analysis (CFA) and the model fitted the data. Satisfactory levels of internal consistency, discriminant and convergent validity were found. (4) Conclusions: The VSS reveals good psychometric properties and can be considered a useful tool for professionals and future research for volunteers’ satisfaction assessment.

## 1. Introduction

Volunteering can be defined as a long-term planned behavior that preferably occurs in an organizational context [1,2,3], involves a non-mandatory form of help [4,5], without any expectation of monetary reward [6], and with people with whom there is no previous connection [5]. Therefore, it is an interesting phenomenon that, for several reasons, should not happen [7]. Investigators wonder about the reasons that lead people to initiate and to maintain volunteer activity. As a response, the motivations that predict and sustain involvement in the form of social action have been examined [5,8,9]. In this context, the literature is based on a functional approach that emphasizes the action purposes and the role of such purposes in initiating, guiding, and sustaining action [10].

Regarding volunteer satisfaction, it can be defined as the pleasure and interest felt by volunteers and the importance and meaning they truly believe their work has for the beneficiaries [11]. It seems to be an important construct that is related to the fulfilment of volunteer motivations. According to Clary and collaborators [8], volunteer satisfaction is influenced by experience, and the more adapted to and satisfied the volunteer is in the organization and tasks developed, the more intention to remain they will reveal. The volunteer process model (VPM) [5,7,12,13] also refers to the importance of the match between motivations and volunteer satisfaction. The VPM emphasizes the dynamic properties of volunteering within and between stages (antecedents, experience, and consequences) and levels of analysis (agency, individual volunteer, and social system). The authors add the importance of expectations in determining volunteers’ satisfaction [13].

Volunteering does not involve any form of monetary reward; however, volunteers often perceive psychological and social costs in their actions, but they continue involved with their volunteering [14]. In the VPM there is a path between satisfaction and sustaining volunteering, but there is a lack of consensus regarding these constructs in the literature. For example, some researchers [5,9,14] found a direct and meaningful path, where others described an indirect one [15] or no path at all between the constructs [16].

In the organizational context, the authors conceptualized organizational integration with two different constructs: organizational commitment and engagement. The first has shown itself to be the best predictor of remaining in volunteering work [17,18] and the way it predicts this intention varies over the period of volunteer work [19]. The model of the two cycles of volunteering developed by Wymer and Starnes [20] highlights that there are two phases that characterize volunteering. The first phase was defined by the authors as the “honeymoon phase”, which is marked by the desire to engage in voluntary work and the resulting gratification. Volunteer satisfaction is a strong predictor of the intention to remain in volunteer work in this phase. The second one starts on average after six months of volunteer work, when knowledge of the negative aspects of the organization counterbalanced its initial idealization. Organizational commitment and role identity seem to be the strongest predictors of the intention to remain after the first six months [15].

Vecina and collaborators [14] propose engagement because it relates positively with workers’ identification with activities and work evaluation as challenging instead of demanding and stressful [21]. There is also a positive relationship with organizational commitment [22]. Another set of reasons is associated with organizational integration. The activities developed in an organizational context and evaluation of their experience of the volunteering environment are important to their sustained work [5,8]. Therefore, work satisfaction is a very important variable in the context of paid work [1] and is even more relevant in the volunteering environment, although non-profit organizations are different from other kinds of organizations [15]. 

Regarding the assessment of satisfaction in volunteering, there are two types of measures. Some studies have one-dimensional measures [6,10] focused on distinct aspects (e.g., tasks, organizational trends), while others consider different dimensions, that result from job satisfaction and other measures that are related (e.g., regarding empowerment, participation efficacy, organization support and group integration [11,23]). Although a single dimension using a single-item measurement is frequently used [24], this type of assessment can limit the understanding that different dimensions can promote. A one-factor instrument can reflect a general satisfaction among volunteers, while a multi-dimensional one may reveal satisfaction related to specific domains, helping us to understand which factors need more attention to maintain volunteers in the activity [25].

One instrument used to assess volunteer satisfaction is the volunteer satisfaction index (VSI) [11] composed of 39 items that assess five main elements (i.e., communication quality, work assignment, participation efficacy, support, and integration). The VSI has been adapted and used in different settings (e.g., Sports [26], Social [27] and Military [28]) and different countries (e.g., China [24,27], Serbia [25] and Russia [29]). However, some researchers, when using the instrument, suggest the omission of several items or only selecting a few items in the original version that are more accurate for evaluation [24,25,26,27].

In 2009, Vecina and collaborators [1] developed a 17-items instrument—the volunteer satisfaction survey (VSS) to assess volunteer motivation according to three dimensions: (1) satisfaction of motivations (based on the volunteer functions inventory [8]); (2) satisfaction with the management of the organization (based on the conceptualization of Penner [30]; and (3) satisfaction with the tasks (based on the job diagnostic survey [31]). The authors analysed the importance of each dimension for the intention to remain and found that volunteer satisfaction accounted for only 8.3% of the volunteers’ intention to continue their work. However, there are few studies that have used and examined the VSS [19,32,33].

Considering that in the Portuguese context there are few studies that use a standardized measurement for assessing volunteer satisfaction, some use a small number of selected items from several scales to fit the criteria of evaluation [34,35] and the importance of volunteering satisfaction. We considered exploring the VSS in the Portuguese context because it is also an instrument that needs further study related to its psychometric characteristics. In this way, the aim of our work was to explore the psychometric properties of the VSS among a sample of Portuguese volunteers, including: (1) VSS internal structure with confirmatory factor analysis; (2) internal consistency; and (3) convergent validity with other constructs (motivation, organizational commitment, and engagement).

## 2. Materials and Methods

### 2.1. Sample

Data were collected from 335 volunteers, for a mean of 44.05 months (*SD* = 65.75, between 1 and 600 months), working mainly weekly (64.2%) in the social (74.7%) and health (17.3%) setting. Their ages ranged from 14 to 81 years old (*M* = 36.26, *SD* = 14.75), and 79 were male (23.6%) and 256 female (76.4%).

### 2.2. Instruments

The volunteer satisfaction survey (VSS) [1] was translated and adapted. This questionnaire is composed of 17 items that assess three subscales: (1) Satisfaction of motivations (six items: e.g., “The tasks I usually perform as a volunteer allow me to establish social relationships with different people”); (2) Satisfaction with the management of the organization (seven items: e.g., “Satisfaction with the overall management of the organization”); and (3) Satisfaction with the tasks (four items: e.g., “I am satisfied with the effectiveness with which I perform the tasks assigned to me”). Responses were given using a seven-point Likert-type scale (1 = Very dissatisfied; 7 = Very satisfied). Dimension and scale scores were computed by averaging items, with higher scores indicating greater satisfaction.

The Utrecht work engagement scale (UWES) [36] was used to assess the volunteers’ engagement. This instrument is composed of 15 items that assess three underlying dimensions: (1) Vigor (five items: e.g., “At my job, I feel strong and vigorous”; α = 0.85); (2) Dedication (five items: e.g., “My job inspires me”; α = 0.85); (3) and Absorption (five items: e.g., “I am immersed in my work”; α = 0.81). Using a seven-point Likert-type scale, participants scored each item ranging from (0 = Never; 6 = Every day). Dimension and scale scores were computed by averaging items, with higher scores indicating greater volunteering work engagement.

The Portuguese Version of the organizational commitment questionnaire (OCQ) [37] was used to evaluate the volunteers’ commitment and attachment to the organization. Three dimensions were assessed: (1) Affective (eight items: e.g., “I am willing to put in a great deal of effort beyond that normally expected in order to help this company to be successful”; α = 0.85); (2) Cognitive (four items: e.g., “I feel little loyalty to this organization”; α = 0.64); (3) and Behavior commitment (three items: e.g., I really care about the fate of this company”; α = −0.45), were assessed on a seven-point Likert-type scale (1 = Strongly disagree; 7 = Strongly agree). The total scale alpha was 0.82, without the behavior commitment subscale. Dimension and scale scores were computed by averaging items, with higher scores indicating greater satisfaction.

A sociodemographic questionnaire was also used to collect information on gender, age, and volunteering-related variables (e.g., organization, area, volunteering dedication).

### 2.3. Procedures

This study was approved by-[Blind to review]. 

The VSS was translated and adapted according to a back-translation process [38] following permission from the original authors.

The data collection was conducted in volunteering organizations (e.g., Food Bank, Portuguese Red Cross) with a snowball sampling technique (students from the Psychology Department of the University of [blind to review] were asked to participate in this study by recruiting five volunteers to complete the questionnaires). At the recruitment phase, all participants were informed about the study aims, its non-compensatory nature, the anonymous and confidential nature of their responses as well as the possibility of withdrawing the study at any time without any negative consequences.

### 2.4. Data Analysis

Data were processed with IBM SPSS 28.0.1.0 (IBM Corp., Chicago, IL., USA), R version 4.2.0 and the *lavaan* package (v0.6-7) [39,40,41]. Missing values were replaced using the EM method as data are MCAR. Descriptive statistics were calculated (e.g., mean, standard-deviation, kurtosis, skewness) and absolute values of skewness bigger than 2 and kurtosis higher than 7 were considered as indicators of violation of the univariate normality assumption [41]. A cut-off value of 3 was considered for multivariate kurtosis.

The factorial structure of the Portuguese version of the VSS was assessed with a confirmatory factor analysis (CFA). When the assumption of multivariate normality was not met, the use of maximum likelihood (ML) or generalized minimum squares estimation methods can affect the quality of the indexes of fit and the estimates of the parameters. Therefore, the robust DWLS (diagonally weighted least squares) estimation method in the *lavaan* (latent variable analysis) package of R was used to account for the non-normal characteristics of the items. 

Goodness of fit indices were obtained, namely: The ratio of the chi-square statistic to the respective degrees of freedom; Comparative fit index (CFI); Goodness of fit index (GFI); Incremental fit index (IFI); Tucker−Lewis fit index (TLI) Root mean square error of approximation (RMSEA); and Standardized root mean square residual (SRMR). Furthermore, a ratio of the chi-square statistic to the respective degrees of freedom less than 2 was considered as an indicator of a good fit; fit index values bigger than 0.95 and RMSEA values less or equal than 0.05 were considered as indicators of a very good fit; and SRMR values less than or equal to 0.08 were considered as indicators of a good fit of the model to the data. Regarding the standardized loading of the items, values bigger than 0.50 were considered as meaningful. The discriminant validity was assessed with the average variance extracted (AVE) and values bigger or equal to 0.5 were considered adequate [42]. Internal consistency was assessed calculating Cronbach alpha, composite reliability (CR), average inter-item correlations, mean inter-item correlation (MIIC) and corrected item-total correlation range (CITCR). Regarding Cronbach alpha, scores above 0.70 were considered as adequate and above 0.90 as excellent [43]. If the score found was below 0.50, we chose to eliminate the subscale. The values of CR bigger than 0.7 also indicated proper reliability of the factors. The recommended range for MIIC is between 0.15 and 0.5 and CITCR values should be above 0.3. 

To analyze associations between the VSS and other scales, Pearson correlations were used.

## 3. Results

### 3.1. Descriptive Analysis

The mean values of the VSS items ranged between a minimum of 4.23 (item 13, *SD* = 2.02) and 5.85 (item 16, *SD* = 1.33), showing dispersion in the answers given to the various items, which is confirmed by the standard deviation with values above 1. The answers ranged between a minimum of 1 and a maximum of 7 and all items registered the possible limits of the response scale.

As can be seen in Table 1, the univariate skewness coefficients are all negative; therefore, all variables have a distribution with negative skewness or asymmetry to the left. As for the kurtosis values, they show a higher number of positive values, which may be considered as a platykurtic distribution (i.e., with a higher degree of flattening than the Normal). Given the reported values, it is considered that there is no severe violation of the univariate normality assumption, but since the multivariate normality assumption was not verified, the method used was DWLS.

### 3.2. Internal Structure Analysis

To assess the model fit, several robust indices of fit were evaluated (Table 2). The overall assessment of the 3-factor structural model [χ^2^(116) = 129.96; χ^2^/*df* = 1.04; CFI = 0.99; TLI = 0.99; RMSEA = 0.01; 90% CI [0.00–0.03] indicates a very good fit of the model to the data. The ratio of the chi-square statistic to the respective degrees of freedom met the recommended criterion (χ^2^/*df* = 1.04) for a good fit of the model to the data. CFI, TLI, RMSEA meet the recommended criterion for a very good fit and SRMR for a good fit.

All estimated factor loadings additionally met the recommended cutoff point of 0.50 as they ranged from 0.69 to 0.84 and therefore showed proper individual reliability values (*R*^2^ ≥ 0.25), indicating factorial validity (Table 3). The z-test values also indicate statistical significance ranging between 14.13 and 19.72.

The results presented in Table 4 indicate acceptable convergent validity of the construct as the average variance extracted (AVE) values for factors F2 and F3 are bigger than or equal to 0.5. 

For F1, the value is less than 0.5, but the composite reliability is higher than 0.6 (Table 5). The correlations between factors range from 0.51 to 0.70. AVE values also indicate discriminant validity as their values are greater than or equal to the square of the values of the correlations between factors.

**Figure 1:**
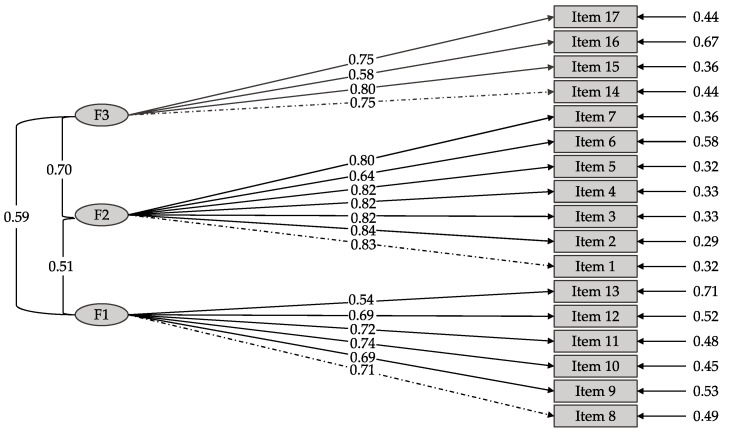
CFA model results.

Figure 1 shows the VSS structural model provided by the CFA. Factor 1 corresponds to satisfaction with the specific motivations, factor 2 to satisfaction with the organization management and factor 3 to satisfaction with the volunteering tasks.

### 3.3. VSS Internal Consistency

Cronbach’s alpha scores are between 0.81 and 0.92, indicating a good internal consistency. The values of the composite reliability (CR) also indicate proper reliability of the factors as all values are bigger than 0.6. The values of the average inter-item correlations range from 0.47 to 0.64. Furthermore, MIIC values for specific motivations and volunteering tasks additionally indicated good internal consistency as they were within the recommended range indicating that their items are sufficiently homogenous to describe the same construct, but that their unique variance distinguishes one from the other, while the organization management subscale’s MIIC value was higher than 0.5, which indicates that items can be a little repetitive. Regarding CITCR, all values range above 0.3 also indicated good internal consistency. 

### 3.4. VSS Convergent Validity

The VSS subscales reveal positive and significative correlations with all engagement subscales (i.e., UWES) and with all the organizational commitment subscales (i.e., affective and cognitive) (Table 6). 

## 4. Discussion

Given the importance of volunteer satisfaction according to several investigations [5,7,8,13], the present study aimed at developing a Portuguese version of the volunteer satisfaction survey (VSS) [44] and evaluating its structure and psychometrics properties. The original version was considered an adequate instrument [44] for assessing volunteers’ satisfaction and other studies reported acceptable psychometric properties [30,31,32].

The distribution of the responses in each item reveals no skewness and kurtosis problems, while the average values are located at the midpoint of the seven-point scale. These indicators were pointed out in the literature as desirable [45]. The normal distribution is suggestive of the discriminative power of the items.

The model assessed fitted the original model by Vecina and colleagues [1]. The three factors are (1) Satisfaction with the specific motivations, (2) Satisfaction with the organization management, and (3) Satisfaction with the volunteering tasks. Therefore, the VSS can contribute to the understanding of the personal functions involved in volunteering [9], from the more self-centered to the more hetero-centered. In addition, it reveals volunteers’ satisfaction with their activities and organizations, and at what point they match with their expectations. According to the VPM, these indicators, when satisfied, support closeness relationships and volunteers’ and clients’ health [13].

The results also showed good reliability and validity index factors. The AVE of the first factor reveals values near 0.5, but because the AVE of the other factors is adequate, the global AVE can be considered acceptable [46]. As expected, the functions evaluated by the VSS showed moderate to strong and significative correlations with each other, and with organizational commitment (not with the behavior component) and psychological engagement [15]. The absence of a significative correlation with the behavior component can be explained by considering organizational commitment. Organizational commitment is one important construct of the VPM, also situated in the experience stage (it is included in the integration domain with psychological engagement), and according to several authors is the best predictor of volunteer sustaining [18,19]. According to the model of the two cycles of volunteering [21], the first phase of volunteering is characterized by the desire of volunteers to engage in their own voluntary work, and by gratification and satisfaction in the work. This phase lasts on average for the first 6 months. In this way, volunteer satisfaction constitutes the strongest predictor of the intention to remain in volunteer work. The second phase is marked by knowledge and awareness of the critical aspects of the organization. In this phase, the strongest predictor becomes role identity [16]. Because our sample is composed of volunteers who were working in volunteering organizations for an average of 44.05 months, satisfaction with volunteering showed a significant relationship with organizational commitment, which can express an intention to remain in the organization.

Considering our initial aim, the VSS results reveal good psychometric properties, and they can constitute a useful and valuable instrument, of easy application and interpretation, to assess volunteers’ satisfaction in organizational and educational settings.

## 5. Conclusions

This study represents a first step in the context of adapting the VSS for the Portuguese population. The first results are positive because the survey adequately satisfies the adopted reliability criteria. However, the effort to adapt and study an instrument must be permanent, which is why the limitations of the present study should be considered and some steps for future research are suggested. The first limitation is the fact that the data are cross-sectional and therefore we cannot produce any longitudinal conclusions, especially regarding the instrument’s relation to permanence in volunteering. In future investigations, it could be relevant to develop longitudinal studies and analyze volunteer satisfaction variance over time. Secondly, the volunteers’ sample used had a broad range of volunteering experience and age, which should be controlled in future studies [3,13]. Finally, the volunteers in the present sample worked in only two contexts (social and health); some studies have shown that there are differences between volunteers’ integration and satisfaction according to the volunteering settings (e.g., social, health, ecological). In future studies it could be pertinent to include volunteers in other contexts. 

The present study presents important conclusions about the psychometric properties of the VSS among Portuguese volunteers and the findings show that it is a useful resource that is valid and reliable for the evaluation of volunteers’ satisfaction. The VSS should be a relevant contribution for researchers, organizations, and intervention professionals. 

## Figures and Tables

**Table 1:** Descriptive Statistics of VSS items.

VSS Items	*M*	*SD*	*S*	*K*
Item 1	5.47	1.22	−0.57	−0.05
Item 2	5.43	1.24	−0.63	0.10
Item 3	5.22	1.28	−0.63	0.28
Item 4	5.20	1.44	−0.77	0.18
Item 5	5.73	1.27	−1.01	0.58
Item 6	4.67	1.69	−0.37	−0.66
Item 7	5.26	1.52	−0.73	−0.06
Item 8	5.78	1.34	−1.22	1.33
Item 9	5.56	1.40	−0.90	0.32
Item 10	4.82	1.79	−0.65	−0.56
Item 11	5.42	1.43	−0.95	0.53
Item 12	4.87	1.83	−0.60	−0.66
Item 13	4.23	2.02	−0.14	−1.24
Item 14	5.71	1.36	−1.20	1.18
Item 15	5.61	1.23	−0.76	0.23
Item 16	5.85	1.33	−1.34	1.46
Item 17	5.79	1.12	−1.01	1.15
Multivariated			61.15	455.52

*Note. M* = Mean; *SD* = Standard deviation; *S* = Skewness; *K* = Kurtosis.

**Table 2:** Goodness of Fit Indices for model of VSI tested with CFA.

Indices	χ^2^/*df*	CFI	TLI	RMSEA	RMSEA 90% CI	SRMR
3-Factor Model	1.04	0.99	0.99	0.01	0.00–0.03	0.06

*Notes.* χ^2^/*df* = Chi-square/degree of freedom; CFI = Comparative Fit Index; TLI = Tucker−Lewis Fit Index; RMSEA = Root Mean Square Error of Approximation; CI = Confidence interval; SRMR = Standardized Root Mean Square Residual.

**Table 3:** Loadings of the VSI tested with CFA.

Factors	Items	Unstandardized	St. Error	z-Value	P (>|z|)	Standardized
F1	Item 8	1.00				0. 71
	Item 9	1.01	0.07	15.34	0.00	0.69
	Item 10	1.38	0.09	15.72	0.00	0.74
	Item 11	1.08	0.07	15.44	0.00	0.72
	Item 12	1.32	0.09	15.60	0.00	0.69
	Item 13	1.13	0.08	14.13	0.00	0.54
F2	Item 1	1.00				0.83
	Item 2	1.04	0.05	19.65	0.00	0.84
	Item 3	1.04	0.05	19.72	0.00	0.82
	Item 4	1.17	0.06	19.27	0.00	0.82
	Item 5	1.04	0.05	19.45	0.00	0.82
	Item 6	1.08	0.06	18.46	0.00	0.65
	Item 7	1.21	0.06	19.28	0.00	0.80
F3	Item 14	1.00				0.75
	Item 15	0.97	0.06	16.49	0.00	0.80
	Item 16	0.75	0.05	14.98	0.00	0.58
	Item 17	0.83	0.05	16.26	0.00	0.75

*Notes.* ST. Error = Standard-Deviation Error, Z-value = Wald statistics, P(>|z|) = *p*-value.

**Table 4:** VSI factors correlation matrix and average variance extracted.

Factor	AVE	Correlation Matrix		
		F1	F2	F3
F1	0.45	1.00		
F2	0.62	0.51	1.00	
F3	0.52	0.59	0.70	1.00

*Notes.* AVE = Average Variance Extracted.

**Table 5:** Cronbach’s alphas, mean inter-item correlations, and corrected item-total correlation ranges.

VSS Sub-Scales	Alpha	CR	MIIC	CITCR
Specific motivations	0.83	0.82	0.47	0.62–0.82
Organization management	0.92	0.92	0.64	0.52–0.69
Volunteering tasks	0.81	0.80	0.52	0.61–0.70

*Notes.* Alpha = Cronbach’s alpha; CR = Composite Reliability; MIIC = Mean Inter-Item Correlation; CITCR = Corrected Item-Total Correlation Range.

**Table 6:** Correlations between VSS, organizational commitment and psychological engagement.

VSS/Other Subscales	OCQ Affective	OCQ Cognitive	OCQ Compromise	UWES Vigor	UWES Dedication	UWE Absorption
Specific motivations	0.45 **	0.20 *	0.45 **	0.36 **	0.37 **	0.27 **
Organization management	0.52 **	0.28 **	0.52 **	0.28 **	0.30 **	0.24 **
Volunteering tasks	0.48 **	0.21 *	0.43 **	0.40 **	0.41 **	0.35 **

*Notes.* OCQ = Organizational commitment; UWES = engagement; * *p* < 0.01; ** *p* < 0.05.

## Data Availability

The data can be made available for consultation upon request to the corresponding author.
